# Informed consent is almost impossible

**DOI:** 10.1002/hem3.70002

**Published:** 2024-09-15

**Authors:** Louise Caldwell, Stephen P. Hibbs

**Affiliations:** ^1^ Barts Health NHS Trust London UK; ^2^ Wolfson Institute of Population Health Queen Mary University of London London UK

Informed consent underpins the practice of medicine. Beyond signing a form, the consent process provides both space and time for patients to gain a better understanding of the practicalities of the treatment and to ask questions of their clinician. If done well, informed consent can enable patient autonomy, facilitate a better understanding of the short‐ and long‐term implications of treatments, and share power with patients in the decision‐making process.

But many of us will have encountered situations where ‘informed consent’ has been recorded, yet a myeloma patient is later surprised and distressed to learn that their chemotherapy was never expected to cure them. A study of over one thousand patients on palliative chemotherapy for lung or colorectal cancer found that the majority did not understand the intent of their treatment.^1^ While our consent practices may be acceptable within legal and bureaucratic norms, how often do patients truly understand the treatments we recommend and prescribe?

Some of the difficulty lies in the approach or capacity of the clinician. The consent process can become rushed, perfunctory and at times transactional. Even the terminology of ‘taking’ consent is telling, evoking a rushed conversation to extract a signature on a consent form so that treatment can commence.

Clinicians often draw arbitrarily sharp lines around decisions that require written consent and those that do not. The *written* component may be less important than the ‘hard stop’ it provides to clinicians, requiring a detailed conversation with the patient before proceeding with treatment. Any chemotherapy—of whatever level of intensity—requires a formal written consent process. In contrast, steroids for immune thrombocytopenia, transfusions in sickle cell disease, or anticoagulation incurs significant risk, but consent practices for these interventions are minimal or inconsistent.^2^

Aside from hurried clinicians, patients face other challenges during consent conversations. In fraught and high‐stakes situations where patients are reeling from a new diagnosis of life‐limiting cancer or are overwhelmed by symptoms of their disease, the intricacies of the treatment they are being consented for may seem marginal or irrelevant. The intensity and immediacy of illness can distort a patient's ability to ‘hear’ the risks of the treatment. Some may approach treatment with complete optimism, suppressing any imagining of risk or failure. Others may be unable to perceive any sort of future whilst confronting an overwhelming present. Can a person ever truly fathom the potential risks that accompany a treatment, when hope demands they believe they will escape them?

Some consent conversations are particularly demanding to both the clinician and the patient. In UK haemato‐oncology practice, patients with a new diagnosis of acute leukaemia are invited to consent to whole‐genome sequencing (WGS) as part of clinical care. The NHS Genomic Medicine Service provides a structured consent form for the purpose of this discussion, in which a patient must understand that WGS is unlikely to affect their treatment, that testing could lead to unexpected findings, and that these findings may have implications for the health of family members.^3^ The patient must understand this while coming to terms with a new acute leukaemia diagnosis, the consent process for intensive chemotherapy, and the possibility of imminent death. They may no longer be alive when the genomic results—including ‘unexpected information’—become available to their families. How often does truly informed consent occur during these complicated, uncertain and distressing conversations?

Are the barriers to truly informed consent possible to overcome? Simple measures can go some distance. Videos explaining the benefits, risks and experience of treatment are an accessible resource for people of any literacy level and can be rewatched ahead of consent discussions, reducing the fear of missing something. And by involving loved ones in consent conversations we acknowledge that life decisions ‐ for all of us ‐ are made through dialogue and relationships with others. Additional barriers encountered by patients with limited language proficiency can be overcome by investment in translation and interpretation. Training for clinicians in consent practices could also be reimagined as something more reflective, responsive to real‐world patient encounters, and ongoing throughout a clinical career. In some other universe, these conversations would be unhurried.

Even then, *genuinely informed* consent at the outset of treatment would sometimes remain unattainable, either because of a patient's emotional or cognitive state, or the complex nature of the treatment or investigation. Without undermining the principle of informed consent, we can acknowledge how difficult it is to achieve through current approaches, even when these meet bureaucratic and legal requirements. If consent is envisaged as a journey, then this creates the opportunity for it to be revisited as a patient comes to terms with both their prognosis and treatment—do we have consent to continue?

Finally, recognising that informed consent is difficult—sometimes almost impossible—challenges the notion that total patient choice is always the priority. Many patients do not view clinicians as dispassionate purveyors of information nor themselves as informed healthcare consumers, but instead seek trustworthy and caring individuals who will remain present with them in their suffering, whatever treatment is pursued.

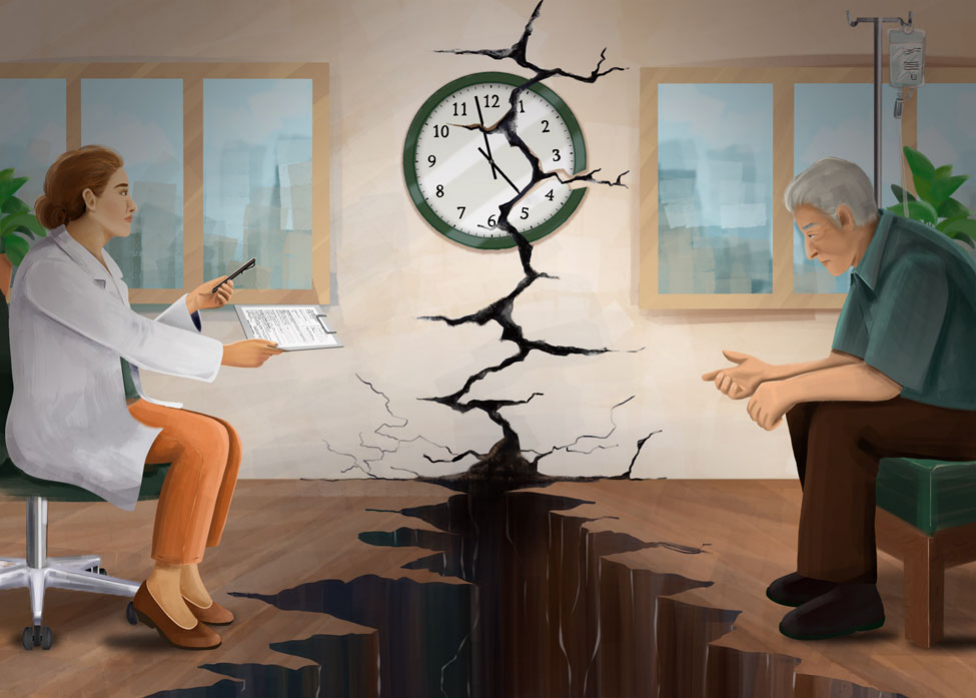

## AUTHOR CONTRIBUTIONS

Both authors conceptualised the article, wrote the initial draft and critically reviewed and revised the article. Both authors agreed to the final version.

## CONFLICT OF INTEREST STATEMENT

The authors declare no conflict of interest.

## FUNDING

Stephen P. Hibbs is supported by a HARP doctoral research fellowship, funded by the Wellcome Trust (Grant number 223 500/Z/21/Z). No funding was received for this publication.

## Data Availability

Data sharing is not applicable to this article as no data sets were generated or analysed during the current study.
